# Address of President Fundenberg

**Published:** 1888-08

**Authors:** 


### ADDRESS OF PRESIDENT W. F. FUNDENBERG.

READ BEFORE THE PENNSYLVANIA STATE SOCIETY MEETING, PHILADELPHIA, JUNE 5, 1888.

The progress made in dentistry during the past half century is marvelous. Instead of a simple ratio, it has assumed a geometrical progression, and has compounded with astonishing rapidity. The advance of the world in new ideas, new subjects, new employments and new discoveries cannot be told in a single address.

Half a century ago primitive modes and means of agriculture were the rule. The few trades which clothed, housed and fed men and the professions of medicine, law and divinity comprised largely all the means and employments ministering to the wants of man. But chemistry, mineralogy, metallurgy, geology, botany, philosophical observation, fine machinery, steam and electricity, have so enlarged the domain of knowledge and the employments of mankind that the province of science and knowledge has assumed an extent unknown to our fathers.

The activity and progress that has distinguished modern medicine has pervaded all its collateral branches. The achievements of surgery, the vast and constantly increasing developments in pathology, the revelations of the microscope, the daily additions to knowledge in chemistry and therapeutics are only part of the general advance along the entire line of scientific research ; and as every collateral branch keeps pace with the march, dentistry has not lagged behind. The science and art of dentistry has shared in the universal progress.

The most condensed review of the progress of dental science, and the improvements in dental practice during the last half century, is not possible within the limit of time permissible on this occasion. It could only be a catalogue of advances—an illustrated record of how new discoveries in general pathology has illuminated the special pathology of the teeth—an outline picture of the fertility of invention in instruments to aid in securing results from operations that were exceedingly difficult if not entirely impracticable—

a meager history of new operations and new methods of operating, and a bare allusion to the ingenuity and genius which has devised the new methods of remedying the many cases of irregularity of the teeth which we are so often called on to treat.

With this advance and improvement in dental practice, the dignity and standing of the dental practitioner has been elevated. The time existed, and not long ago, when a dentist was looked upon in nearly the same light with the "Cupper and Leecher," or if held in higher esteem, as at most only a sort of mechanic, whose business did not require more than a very moderate share of mechanical ingenuity, and who certainly was not supposed to require any knowledge of the vital functions in order to practice his calling in a proper manner.

Gradually dentistry began to take higher rank. Many men of education entered its ranks, and imparted to it tone and character, and before long it was conceded that dentistry was both an art and a science, and its practice no longer a trade, but an honorable profession. Then dental schools were established, at first weak, unrecognized and ridiculed ; but in time they grew in strength and number as the importance and needs of special dental education became recognized.

The subject of dental education has been so often discussed before you that were I to occupy your time with a full presentation of its necessities I would, I fear, only weary you with a repetition of remarks with which you are already familiar. The importance of a firm foundation being first laid in the preliminary education of the student is acknowledged by all, and the best means of effecting this, and the standard which shall be required of all who essay to enter the profession, is a question for consideration. The elevation of dental schools in the standard required of students, the thoroughness of the course of study, and the rigidness of final examinations demands the attention of the profession. The profession needs better matriculants and better graduates in dentistry.

Much good has been obtained by special dental education and restrictive dental legislation. The law requiring every dentist to be registered has worked much good to the profession and to the community; but it yet requires increased powers. Where the law has been enforced it has driven away and excluded many unqualified practitioners. There is a difficulty in enforcing the law—by getting a jury to indict a violator. To the Pittsburg Dental Society belongs the credit of having obtained the first conviction under our present law, which occurred within the past month.

But we need a better law, simpler and more restrictive; for the simpler and plainer the law the more easily it can be enforced. A law requiring every one who shall hereafter engage in the practice of dentistry to be a graduate of a reputable dental school, and the registration of his diploma endorsed by a board appointed by the State Society, would be more effective. Such a law would not exclude any one who is at present practicing legitimately.

The wording of a bill to meet the case is a matter demanding consideration. Experience teaches the importance of carefully determining, in advance, what and how much power the legislature can be induced to give. The practical question in the beginning of such attempted legislation is not so much what we would like to have as what can be gotten. It is easy to defeat a measure by asking too much.

Public sentiment must be educated to the need of protective dental legislation. The people and their representatives must be taught that strict State supervision of dental practice is not class legislation—that dental legislation is as much a protection to the people as to the profession.

In the daily interchange of opinions with legislators and constituents, the members of this society and of the profession could exert a widespread influence in shaping public opinion and in securing a satisfactory dental law.

The influence that dental societies exert in the advancement of dentistry and in the elevation of the profession is underestimated by many.

The increase in the numbers of district societies should be a matter for consideration by the society.

Whatever advances have been made in the State, have either been urged upon the profession and the schools by this body, or have received the endorsement of this society when inaugurated.

The State Society has accomplished much good since its organization. It has been the prime mover in the past—we may hope that it will continue so in the future.

